# Hepatocyte-Targeted Drug Delivery Strategies for Chronic Hepatitis B: Overcoming Delivery Barriers Toward Functional Cure

**DOI:** 10.3390/pharmaceutics18020212

**Published:** 2026-02-07

**Authors:** Ayman Elbehiry, Musaad Aldubaib

**Affiliations:** 1 Department of Public Health, College of Applied Medical Sciences, Qassim University, Buraydah 51452, Saudi Arabia; ar.elbehiry@qu.edu.sa; 2 Department of Pathology and Laboratory Diagnosis, College of Veterinary Medicine, Qassim University, Buraydah 51452, Saudi Arabia

**Keywords:** hepatitis B virus, hepatocyte targeting, drug delivery, covalently closed circular DNA, functional cure, nucleic acid therapeutics

## Abstract

Chronic hepatitis B remains difficult to cure because viral persistence is maintained within hepatocytes through covalently closed circular DNA and integrated viral sequences that continue to drive antigen production even when viral replication is effectively suppressed. Although current antiviral therapies improve clinical outcomes and slow disease progression, they rarely achieve a durable functional cure, defined as sustained loss of hepatitis B surface antigen (HBsAg), with or without anti-HBs seroconversion. This limitation has shifted attention toward therapeutic strategies that depend on precise and reliable drug delivery to the liver. Several recent reviews have focused on antiviral mechanisms or immune modulation. However, the specific contribution of drug delivery to therapeutic success has not been systematically addressed. This review examines hepatocyte-targeted drug delivery as a central determinant of success for emerging hepatitis B therapies. Rather than cataloging individual therapeutic agents, this review adopts a delivery-centered framework that links viral persistence biology with translational feasibility across therapeutic classes. Recent advances in ligand-mediated hepatocyte targeting have demonstrated consistent liver specificity and clinical feasibility, enabling meaningful reductions in viral transcripts and antigens. At the same time, we discuss why more complex delivery platforms continue to face challenges related to intracellular access, immunogenicity, scalability, and safety during repeated dosing, particularly for strategies intended to act within the nucleus. Translational and clinical considerations, including differences between experimental models and human infection, manufacturing and regulatory constraints, and the demands of long-term treatment, are also addressed. Overall, this review supports a pragmatic path toward functional cure based on rational combination therapies, coordinated delivery strategies, and patient-tailored approaches, with delivery science serving as the critical link between biological insight and durable clinical benefit.

## 1. Introduction

Chronic hepatitis B remains a major cause of cirrhosis and hepatocellular carcinoma worldwide, despite the availability of effective vaccines and potent antiviral drugs. The World Health Organization estimates that hepatitis B caused about 1.1 million deaths in 2022, mainly due to cirrhosis and primary liver cancer [1]. In the same year, viral hepatitis overall caused approximately 1.3 million deaths, with hepatitis B accounting for most of this burden [2,3]. These figures highlight a central paradox. Prevention is highly effective, yet the global disease burden persists. This persistence reflects the large population already living with chronic infection and remaining at risk of progressive liver disease [3].

Current antiviral therapy can suppress circulating viral replication and improve clinical outcomes. However, it rarely achieves an endpoint that allows safe treatment discontinuation. Clinical guidelines consistently recommend nucleos(t)ide analogs, including entecavir, tenofovir disoproxil fumarate, and tenofovir alafenamide, because of their high antiviral potency and low resistance rates [4,5].

Many patients, particularly those with HBeAg-negative immune-active disease, require long-term therapy. Treatment withdrawal is frequently followed by viral rebound and inflammatory flares [4]. This clinical pattern reflects the biological resilience of hepatitis B virus within hepatocytes. It also explains why long-term viral suppression has not translated into population-level elimination of advanced liver disease [1,2]. For example, long-term nucleos(t)ide analog therapy effectively suppresses serum HBV DNA but does not directly target intranuclear viral templates. As a result, treatment discontinuation is followed by viral rebound in the majority of patients [4,6].

Major guidance documents describe functional cure as sustained loss of hepatitis B surface antigen. This endpoint is usually accompanied by persistently suppressed serum hepatitis B virus DNA after a finite course of therapy. Here, a finite course refers to treatment discontinuation following achievement of sustained off-treatment viral suppression, while acknowledging that relapse risk remains an active area of investigation [4,7]. This endpoint is clinically important because hepatitis B surface antigen (HBsAg) loss is associated with a markedly improved prognosis.

Nevertheless, ongoing surveillance may still be required in some patients. The risk of hepatocellular carcinoma can persist, particularly in those with cirrhosis or advanced age at antigen loss [4]. The low frequency of HBsAg loss with nucleos(t)ide analog therapy therefore drives current research efforts toward strategies that reduce antigen burden, disrupt viral persistence, and restore durable immune control [7,8].

Emerging approaches include hepatocyte-targeted nucleic acid-based therapies, such as small interfering RNAs delivered via N-acetylgalactosamine (GalNAc) conjugation. These strategies have demonstrated clinically meaningful reductions in viral transcripts and HBsAg levels. However, repeated dosing is generally required to maintain effect [9,10].

Functional cure remains uncommon because viral persistence is maintained within infected hepatocytes. Covalently closed circular DNA (cccDNA) serves as a stable nuclear template for viral transcription. It continues to support viral protein production even when serum hepatitis B virus (HBV) DNA becomes undetectable during therapy [7,8].

In parallel, integrated HBV DNA can contribute to ongoing antigen expression. This persistent antigen production may sustain immune dysfunction and complicate attempts to reestablish effective antiviral immunity [7]. These features distinguish hepatitis B from infections in which pathogen clearance can be achieved simply by suppressing circulating replication. They also place hepatocyte-level targeting at the center of the therapeutic challenge [8].

For instance, therapies that act extracellularly or at the hepatocyte surface, such as entry inhibitors targeting the sodium taurocholate cotransporting polypeptide, prevent new infection events. However, they do not eliminate pre-existing intracellular viral reservoirs [11,12].

Several recent reviews have focused on antiviral mechanisms, immune modulation, or individual therapeutic classes. In contrast, the contribution of drug delivery to therapeutic success has been less systematically examined across modalities. At this point, drug delivery becomes decisive rather than auxiliary.

Many emerging therapies depend on efficient hepatocyte targeting, access to specific intracellular compartments, and sufficient intracellular activity while limiting systemic exposure. The growing emphasis on functional cure in contemporary guidance indicates that future treatment will likely rely on combinations of direct-acting antivirals and immune-directed approaches [5,7]. These strategies increase the importance of tissue specificity, intracellular trafficking, and tolerability under repeat dosing.

Delivery challenges are amplified in the liver because hepatocytes are surrounded by non-parenchymal cells that readily sequester therapeutic systems. In addition, repeated administration must be compatible with long-term safety in a chronic disease setting [8]. Clinical experience with liver-directed delivery platforms illustrates this challenge. Efficient hepatic accumulation does not necessarily translate into productive hepatocyte uptake or nuclear access required for durable antiviral effects [13,14].

Accordingly, this review adopts a delivery-centered perspective that links viral persistence biology with clinical and translational feasibility across therapeutic classes. Rather than providing an inventory of therapeutic agents, we focus on how hepatocyte targeting, intracellular trafficking, and nuclear access shape the real-world potential of emerging strategies.

This narrative review treats drug delivery as a core determinant of whether functional cure strategies can succeed in humans. The next section outlines the microbiological basis of viral persistence despite potent antiviral suppression, with emphasis on cccDNA and persistent antigen expression [7,8]. The review then examines hepatocyte-specific biological barriers that shape intrahepatic distribution and intracellular trafficking before presenting principles that guide hepatocyte-focused targeting [8].

**Figure 1:**
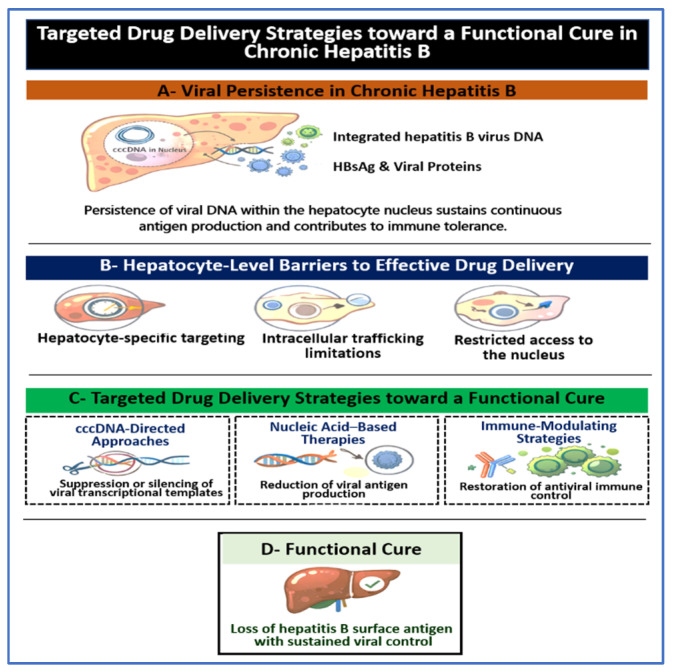
Conceptual framework linking viral persistence, hepatocyte-level delivery barriers, and targeted drug delivery strategies in chronic hepatitis B. (**A**) Viral persistence is sustained by covalently closed circular DNA and integrated viral DNA within the hepatocyte nucleus, which drive continued viral antigen production and contribute to immune tolerance. (**B**) These persistence mechanisms create hepatocyte-level barriers that limit antiviral efficacy, including challenges related to selective hepatocyte targeting, inefficient intracellular trafficking, and restricted access to the nucleus. (**C**) Targeted drug delivery strategies address these barriers through systems designed to suppress viral transcriptional templates, reduce antigen production via nucleic acid–based therapies, and restore antiviral immune control. (**D**) Integration of biological targeting and delivery strategies aims to achieve functional cure, defined by loss of HBsAg with sustained viral control.

Finally, targeted delivery approaches are evaluated according to therapeutic intent across the major classes shaping the current treatment strategies, with attention to translational constraints that determine whether a delivery concept can progress from experimental promise to clinical use [5]. Figure 1 provides a conceptual overview of viral persistence barriers in chronic hepatitis B and the delivery requirements that must be addressed to align emerging therapies with the goal of functional cure.

## 2. Viral Persistence in Chronic Hepatitis B: A Microbiological Foundation

HBV persists because it establishes durable viral DNA within the nuclei of infected hepatocytes and continues to produce viral antigens even when circulating viremia is pharmacologically suppressed. Nassal first identified covalently closed circular DNA (cccDNA) as the central molecular reservoir that sustains the HBV life cycle and represents a major barrier to curative therapy [15]. Zoulim later emphasized that cccDNA resides in the hepatocyte nucleus and serves as the transcriptional template for all viral RNAs, making it a primary target for cure-directed strategies [16].

The nuclear localization and long-term stability of cccDNA represent a major therapeutic limitation. This episome is physically shielded from many antiviral agents and can persist for years in nondividing hepatocytes [17,18].

Following entry into hepatocytes, the incoming relaxed circular DNA genome must be converted into cccDNA before productive viral transcription can occur. Wei and Ploss described the mechanistic steps involved in cccDNA formation and highlighted that conversion from relaxed circular DNA (rcDNA) to cccDNA is a critical early event that depends on host-mediated processing and repair [19]. They further showed that distinct lesions on the minus and plus strands of rcDNA are repaired by different host DNA repair factors. This finding supports the concept that cccDNA formation relies on cellular repair pathways rather than virus-encoded enzymes [20].

More recently, Gómez-Moreno and Ploss characterized cccDNA as a chromatinized viral minichromosome that forms from incoming viral DNA and persists as the dominant template for HBV gene transcription [21]. Because cccDNA formation and maintenance depend on host DNA repair and chromatin-regulating pathways, therapeutic interference is constrained by the risk of disrupting essential cellular functions [22,23].

Once established, cccDNA functions as a stable nuclear episome that can remain transcriptionally active for extended periods. This stability allows continued synthesis of viral RNAs and proteins. Nassal explained that this transcriptional capacity enables persistent viral protein production even when downstream replication steps are inhibited, accounting for the limited curative effect of standard antiviral therapies [15].

Subsequent work reviewed by Hong and colleagues showed that cccDNA transcription is regulated epigenetically and depends on host RNA polymerase II, reinforcing the reliance of HBV on host nuclear machinery [24]. Although transcriptional silencing of cccDNA is conceptually attractive, current epigenetic approaches often lack durability and specificity, limiting their ability to achieve sustained viral control [25].

This biology explains why nucleos(t)ide analogs, despite their high efficacy in suppressing serum HBV DNA, rarely achieve durable off-treatment control. Nassal described cccDNA as the persistence reservoir that remains after inhibition of reverse transcription, resulting in long-term viral suppression without true cure [15]. Zoulim later noted that current therapies do not directly eliminate the nuclear cccDNA pool and therefore do not remove the transcriptional source of viral RNAs and antigens [16].

Clinical studies consistently show that viral rebound occurs in most patients after nucleos(t)ide analog discontinuation, underscoring the inability of replication-targeted therapy to eradicate nuclear viral reservoirs [26,27].

In addition to episomal persistence, HBV frequently integrates fragments of its DNA into the host genome, creating an additional and clinically relevant source of viral gene expression. Tu and colleagues first reviewed the molecular mechanisms and clinical implications of HBV DNA integration and showed that integrated viral DNA can support replication-independent antigen expression, with relevance to disease progression and hepatocellular carcinoma [28].

Bousali and colleagues later summarized evidence that integration events can disrupt host genes involved in cell proliferation and oncogenic pathways. They underscore that integration contributes to long-term liver pathology rather than serving only as a marker of chronic infection [29]. Integrated HBV DNA constitutes a stable and irreversible source of viral antigen expression that cannot be eliminated by current antiviral or cccDNA-targeted approaches [30,31].

Integrated HBV DNA is particularly important because it can sustain HBsAg production even when productive viral replication is strongly suppressed. Ringlander and colleagues reported that integrated viral DNA represents a significant source of HBsAg in humans, suggesting that antigen decline may be limited if integrated templates are not addressed [32].

More recent reviews further linked integrated HBV DNA to ongoing antigen expression and attenuation of antiviral immune responses, connecting integration to both viral persistence and immune dysfunction [33]. Clinical observations indicate that suppression of serum HBV DNA does not reliably correlate with reductions in hepatitis B surface antigen, reflecting ongoing transcription from cccDNA and integrated templates [34].

Persistent antigen exposure shapes the immune phenotype of chronic hepatitis B. Virus-specific immune responses are often impaired rather than sterilizing. Early work described virus-specific T cell dysfunction as an exhaustion state marked by reduced effector function and sustained expression of inhibitory receptors [35]. Subsequent studies showed that secreted viral antigens act as major obstacles to effective antiviral immunity and that HBsAg directly suppresses immune function [36].

More recent analyses confirmed that sustained antigen exposure contributes to immune dysfunction and that antigen reduction is required for immune recovery [37]. Even with partial antigen reduction, restoration of virus-specific immune responses is often incomplete, highlighting a key limitation of strategies that do not sufficiently reduce intrahepatic antigen load [38].

Overall, cccDNA persistence, HBV DNA integration, and sustained antigen production explain why chronic hepatitis B cannot be cured by replication-suppressing therapies alone. Zoulim emphasized that elimination or durable silencing of cccDNA is central to cure strategies, while integrated viral DNA introduces additional transcriptional templates that may also need to be addressed to achieve meaningful antigen loss [16,33].

This microbiological foundation establishes the delivery challenge discussed in the next section. Therapies designed to silence nuclear templates, reduce antigen production, or modulate intrahepatic immunity must reach hepatocytes efficiently and act within specific intracellular compartments to achieve durable clinical benefit [15,21]. These biological constraints indicate that inadequate delivery to the correct hepatic and intracellular compartments is likely to remain a major determinant of clinical failure, even for mechanistically potent antiviral or immune-based strategies [18].

**Figure 2:**
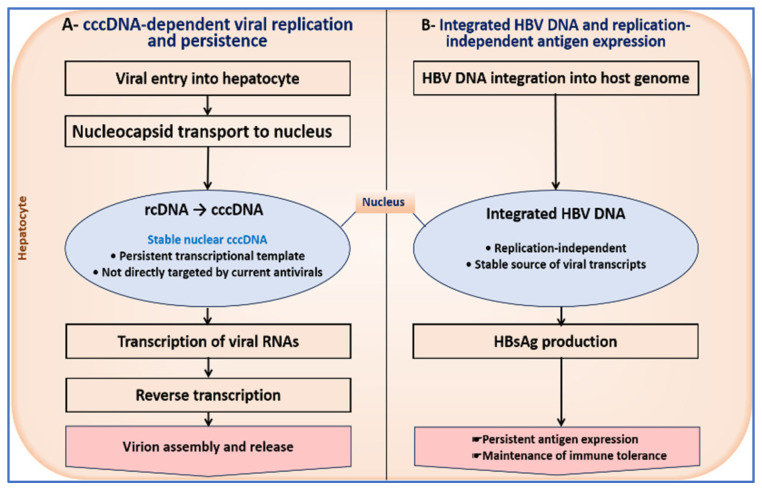
Distinct mechanisms supporting HBV persistence in hepatocytes. (**A**) cccDNA-dependent viral replication and persistence. Following viral entry into the hepatocyte, the nucleocapsid is transported to the nucleus, where relaxed circular DNA is converted into covalently closed circular DNA. Nuclear cccDNA persists as a stable transcriptional template that is not directly targeted by current antiviral therapies and supports transcription of viral RNAs, reverse transcription, and subsequent virion assembly and release. (**B**) Integrated HBV DNA and replication-independent antigen expression. Integration of HBV DNA into the host genome generates stable viral sequences within the nucleus that serve as a replication-independent source of viral transcripts, supporting continued HBsAg production and contributing to persistent antigen exposure and maintenance of immune tolerance during chronic infection.

Figure 2 summarizes the HBV life cycle with emphasis on nuclear viral persistence, highlighting the roles of cccDNA and integrated viral DNA in sustaining antigen production despite effective suppression of circulating viral replication.

## 3. The Hepatocyte as a Therapeutic Target: Biological and Physiological Barriers

### 3.1. Liver Architecture and Exposure of Therapeutics Within the Sinusoid

Systemically administered therapeutics reach the liver through a highly perfused vascular network. Blood flows through sinusoids before entering the hepatocyte-rich parenchyma. DeLeve described liver sinusoidal endothelial cells as a specialized endothelium lining these channels. She emphasized that their structural and functional properties position them as both a gateway and a regulatory interface between the circulation and hepatocytes [39].

As a result, particles and macromolecules within the sinusoidal lumen encounter endothelial and immune scavenger systems before they can access hepatocytes. This anatomical arrangement favors early interception of many delivery systems. More recent analyses reported that sequestration by Kupffer cells and liver sinusoidal endothelial cells often limits the fraction of lipid nanoparticles (LNPs) that reach hepatocytes, even when hepatocytes are the intended target [40]. Consequently, high liver accumulation does not necessarily indicate effective hepatocyte delivery. This represents a major limitation for systemically administered particulate therapies.

### 3.2. Liver Sinusoidal Endothelial Fenestrations as a Selective Gateway

The sinusoidal endothelium is characterized by fenestrations. These pores facilitate exchange between blood and the space of Disse and lack a classical basement membrane. Early ultrastructural studies by Braet and Wisse showed that the size and distribution of liver sinusoidal endothelial fenestrae influence access to hepatocytes [41].

Later work confirmed that fenestrations are patent pores within thin endothelial extensions and play a central role in hepatic filtering function [42]. These features enable efficient physiological exchange. At the same time, they impose size- and property-dependent filtering of delivery systems. As a result, hepatocyte exposure may differ substantially from plasma pharmacokinetics (PKs).

DeLeve further noted that liver sinusoidal endothelial cells exhibit high endocytic activity and actively participate in uptake and clearance [39]. This activity can further reduce delivery to hepatocytes. This selective filtering represents a structural limitation for larger or rigid delivery systems. Such systems may be excluded from parenchymal access despite favorable systemic PK profiles.

### 3.3. Competition for Uptake by Non-Parenchymal Cells

A major barrier to hepatocyte delivery arises from competition with non-parenchymal liver cells. Kupffer cells and liver sinusoidal endothelial cells efficiently capture particulate materials from the circulation. Early quantitative observations discussed by DeLeve showed that administration of poly(lactic-co-glycolic acid) NPs in mice resulted in uptake by most Kupffer cells and liver sinusoidal endothelial cells, with substantially lower uptake by hepatocytes [39].

Subsequent reviews confirmed that NPs are predominantly sequestered by Kupffer cells, with additional uptake by other sinusoidal cell populations. This process limits access to parenchymal targets [43]. Tavares and colleagues further demonstrated that the liver can sequester a large fraction of administered nanomaterials. These findings establish hepatic interception as a dominant determinant of NP biodistribution [44].

This competition for uptake is not restricted to a single delivery platform. It is strongly influenced by particle size, surface properties, and protein adsorption in blood. Poon and colleagues demonstrated that both Kupffer cells and liver sinusoidal endothelial cells contribute to size-dependent hepatobiliary processing of gold NPs. This work highlights the coordinated role of multiple hepatic cell types in NP clearance [45].

More recent reviews summarized how NP physicochemical characteristics shape liver interactions. They also described strategies aimed at modulating Kupffer cell uptake to improve delivery efficiency [46]. In the context of chronic hepatitis B, this biology is critical. Delivery systems intended to reach hepatocytes may instead be diverted to hepatic scavenger compartments. This diversion reduces intracellular exposure at the intended site of action.

This limitation has been highlighted for LNPs. In these systems, sequestration by Kupffer cells and liver sinusoidal endothelial cells can substantially restrict hepatocyte delivery [40]. Thus, scavenger cell uptake represents a fundamental efficiency loss. This loss cannot be fully overcome by increasing systemic dose without raising safety concerns.

### 3.4. Hepatocyte Heterogeneity and Zonation

Even when delivery systems reach hepatocytes, hepatocytes do not represent a uniform population. Functional heterogeneity influences drug uptake, metabolism, and intracellular response. Foundational work reviewed by Cunningham and Porat-Shliom described hepatic zonation as a spatial organization of hepatocyte metabolism. Distinct regions along the porto-central axis exhibit different metabolic and physiological programs [47].

Subsequent single-cell and spatial analyses refined this understanding. These studies confirmed that hepatocyte phenotypes vary across microanatomical regions [48]. This heterogeneity has direct implications for targeted delivery. Uptake efficiency, intracellular metabolism, and toxicity can differ by zone.

As a result, average hepatic exposure may not reflect exposure in hepatocyte subpopulations most relevant to HBV persistence. Cunningham and Porat-Shliom emphasized that spatial organization of metabolism is fundamental to liver physiology. This supports inclusion of zonation in interpretation of delivery and pharmacodynamic data [47]. This zonation introduces variability in therapeutic response and complicates dose–response interpretation for hepatocyte-targeted strategies.

### 3.5. Intracellular Trafficking Barriers and Restricted Access to the Nucleus

Reaching hepatocytes is necessary but insufficient for therapies whose targets reside in specific intracellular compartments. Many advanced HBV strategies require cytosolic activity, such as gene silencing. Others require nuclear access, such as approaches targeting cccDNA. Intracellular trafficking therefore represents a central barrier.

Early image-based analyses showed that LNP-delivered small interfering RNA undergoes complex intracellular trafficking. These studies also showed that endosomal escape is a limiting step for functional delivery [13]. Later studies confirmed that endosomal escape is quantitatively inefficient for nucleic acid therapeutics. Only a small fraction of internalized cargo reaches the cytosol in vivo [49]. Recent work similarly identified endosomal escape as a major bottleneck for LNP-mediated delivery. This limitation often dominates overall efficiency despite robust cellular uptake [50].

These intracellular constraints are particularly relevant for chronic hepatitis B because the most durable viral reservoir resides in the nucleus. The pathway from endosomal uptake to nuclear access involves multiple sequential barriers. Endosomal retention limits cytosolic availability of therapeutic nucleic acids. Subsequent trafficking and nuclear entry further restrict access to nuclear targets associated with cccDNA.

Quantitative observations support the conclusion that improving intracellular routing, rather than tissue targeting alone, is often required to translate delivery into biological effect [13]. Accordingly, intracellular trafficking inefficiency represents a dominant limitation even for delivery systems that successfully reach hepatocytes.

### 3.6. Implications for Hepatocyte-Targeted Delivery in Chronic Hepatitis B

Liver microanatomy, competition among hepatic cell types, hepatocyte heterogeneity, and intracellular trafficking barriers define a sequence of obstacles. Any cure-directed strategy must overcome these barriers to act in the correct cell and compartment. Early descriptions of the endocytic capacity of liver sinusoidal endothelial cells and the high uptake by Kupffer cells explain why many particulate systems accumulate in the liver without achieving efficient hepatocyte targeting [39,43].

The practical implication is that therapeutic success depends not only on liver delivery. A meaningful fraction of the administered dose must reach hepatocytes. It must escape intracellular sequestration and, when required, access nuclear targets relevant to HBV persistence. Analyses by Gilleron and Dowdy reinforce this view. Their work shows that intracellular handling can remain the dominant limitation even after successful delivery to the target tissue [13,49].

**Figure 3:**
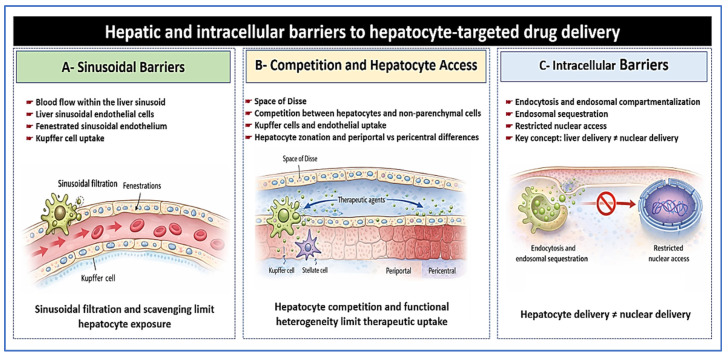
Hepatic and intracellular barriers that limit effective hepatocyte-targeted drug delivery. (**A**) Systemically administered therapeutics enter the liver through the hepatic sinusoids, where blood flow dynamics, the fenestrated sinusoidal endothelium, and rapid uptake by liver sinusoidal endothelial cells and resident Kupffer cells restrict hepatocyte exposure. (**B**) Within the space of Disse, competition between hepatocytes and non-parenchymal cells further limits access to parenchymal targets, while functional heterogeneity arising from hepatocyte zonation results in spatial differences in uptake, intracellular metabolism, and therapeutic response. (**C**) After hepatocyte entry, internalized agents are frequently sequestered within endosomal compartments, and restricted nuclear access limits delivery to intracellular targets relevant to viral persistence. Together, these sequential barriers explain why successful delivery to the liver or hepatocyte does not necessarily translate into effective delivery to the hepatocyte nucleus.

Failure at any step in this delivery sequence can negate otherwise potent antiviral or immune-directed mechanisms. This barrier-based framework provides the rationale for the next section. That section outlines principles guiding hepatocyte-focused targeting and selection of delivery strategies in chronic hepatitis B [40,46]. Figure 3 presents an integrated view of hepatic and intracellular barriers that limit effective delivery to hepatocytes.

## 4. Principles Guiding Hepatocyte-Targeted Drug Delivery for Chronic Hepatitis B

### 4.1. Rationale for Hepatocyte-Specific Targeting

Delivery of a therapeutic agent to the liver is necessary but not sufficient to achieve a cure-directed effect in chronic hepatitis B. Effective therapy requires engagement of infected hepatocytes at adequate intracellular concentrations. It also requires activity within the appropriate cellular compartment.

Clinical experience with patisiran and the widespread use of GalNAc conjugation for liver-directed oligonucleotides demonstrate that durable pharmacological effects depend on efficient hepatocyte uptake and productive intracellular delivery rather than liver exposure alone [51,52]. More recent experimental studies further support this principle. These studies show that delivery systems combining multiple targeting features, such as GalNAc ligands with cell-penetrating elements, can improve hepatocyte uptake and gene silencing compared with single-ligand approaches [53].

However, hepatocyte-specific targeting alone does not guarantee functional activity. Intracellular sequestration and limited access to cytosolic or nuclear targets can still restrict therapeutic efficacy despite efficient uptake.

In hepatitis B, the most relevant therapeutic targets reside in the hepatocyte cytosol and nucleus. Delivery strategies that increase the fraction of the administered dose reaching hepatocytes are therefore essential. Strategies must also support effective intracellular release to convert viral suppression into sustained antigen reduction [14,52].

Targeting via the asialoglycoprotein receptor continues to evolve. Recent advances in ligand design have improved receptor affinity and selective endocytosis. These advances enhance delivery precision for hepatocyte-directed therapeutics [54]. At the same time, reliance on a single receptor pathway introduces vulnerability. Receptor saturation and disease-related variability in receptor expression may limit consistent delivery.

### 4.2. Ligand-Mediated Targeting of Hepatocytes

Receptor-mediated uptake through the asialoglycoprotein receptor is currently the most established and clinically validated strategy for selective hepatocyte delivery. Foundational studies showed that multivalent GalNAc conjugation binds this receptor with high affinity. This interaction supports efficient endocytosis into hepatocytes and enables robust RNA interference after subcutaneous administration [55,56].

GalNAc-conjugated small interfering RNAs and antisense oligonucleotides now underpin several approved and late-stage therapeutics, including inclisiran. These agents provide a practical framework for hepatocyte targeting in hepatitis B [57,58].

Translational studies and clinical experience show that GalNAc conjugation provides consistent hepatocyte selectivity. Predictable pharmacological behavior has been observed across different oligonucleotide chemistries and disease contexts [13,54]. Experimental data also indicate that receptor-mediated uptake has finite capacity. At pharmacologically relevant doses, uptake remains efficient. At very high doses, receptor recycling limits can be transiently exceeded [59].

More recent ligand architectures, including geometry-controlled multivalent displays and peptide-based scaffolds, have demonstrated improved selectivity and uptake in preclinical models [54,60]. These observations suggest opportunities to enhance potency while limiting off-target exposure. They also highlight a limitation of ligand-based systems. Increasing dose does not proportionally increase intracellular activity once receptor-mediated uptake becomes saturated.

Despite these advantages, ligand-based strategies impose important constraints. They require sustained receptor expression and balanced ligand affinity to avoid saturation [52,55]. They also depend on intracellular trafficking routes compatible with the therapeutic mechanism.

In hepatitis B, this includes the need for endosomal escape or nuclear access, depending on the target [56,61]. PK and PD analyses further show that ligand design, dosing interval, and oligonucleotide stability determine hepatic exposure and duration of effect [59]. Because GalNAc-mediated uptake delivers cargo into endosomal compartments, effective therapy must incorporate mechanisms that enable intracellular release when cytosolic or nuclear activity is required [13,62]. Failure to address endosomal escape remains a key reason why robust hepatocyte uptake does not always translate into durable antiviral effects.

### 4.3. Physicochemical Determinants of Hepatocyte Uptake

Particle size, surface chemistry, and charge strongly influence whether delivery systems traverse sinusoidal endothelial fenestrae. These properties also affect early sequestration. LNPs preferentially accumulate in the liver because of their size, composition, and interactions with circulating proteins. Smaller conjugates rely primarily on receptor binding to achieve hepatocyte specificity [46,63].

Quantitative analyses indicate that particles larger than approximately 100–150 nm show reduced access to the parenchyma under physiological conditions [64,65]. Improved control of particle size distribution has therefore emerged as a practical strategy to enhance reproducible hepatocyte exposure in preclinical studies [66]. Nevertheless, size optimization alone cannot prevent uptake by Kupffer cells. It also cannot guarantee delivery to the intended intracellular compartment.

Protein adsorption onto NP surfaces further shapes biodistribution. This process promotes recognition by Kupffer cells and other scavenger systems. Delivery platforms must therefore account for protein corona formation to improve hepatocyte bioavailability [67,68]. Recent studies show that modifying surface chemistry or preconditioning particles can shift uptake away from non-parenchymal compartments and toward hepatocytes without altering the core carrier structure [67,69]. These strategies introduce additional formulation complexity. They may also affect stability, scalability, or reproducibility.

Surface charge and ionizability also affect circulation behavior and intracellular processing. Neutral or ionizable surfaces reduce nonspecific clearance. Strongly cationic materials increase toxicity risk [46,63]. Ionizable lipids illustrate this balance. They remain neutral in circulation but become protonated within endosomes. This change promotes membrane interaction and intracellular release.

While this property contributes to therapeutic activity, it requires careful optimization to limit inflammatory responses [50,70]. Repeated dosing studies further indicate that some surface modifications, including polyethylene glycol coatings, may induce immune responses over time. This is particularly relevant for chronic hepatitis B therapy [46].

### 4.4. Strategies to Reduce Off-Target Uptake by Non-Parenchymal Cells

Kupffer cells and liver sinusoidal endothelial cells capture a substantial fraction of particulate delivery systems. Successful hepatocyte targeting, therefore, often depends on reducing nonspecific recognition or promoting selective uptake. Common strategies include transient surface shielding to reduce opsonization. Control of particle size to favor parenchymal access is also used. Incorporation of ligands that preferentially engage hepatocyte receptors represents another approach [67,71].

Translational decisions must balance immune interactions, manufacturability, and tolerability during repeated administration. For example, polyethylene glycol can reduce early clearance. However, it may provoke immune responses with repeated dosing. Modulation of surface chemistry to influence protein adsorption offers an alternative route to improve hepatocyte delivery [67,71].

### 4.5. Intracellular Trafficking Considerations for Functional Delivery

After uptake of hepatocytes, intracellular trafficking determines whether a therapeutic reaches its site of action. Many hepatitis B strategies require cytosolic or nuclear activity. Intracellular routing is therefore a critical determinant of efficacy. Early imaging studies showed that LNP-delivered small interfering RNA undergoes complex intracellular processing. These studies also demonstrated that escape from endosomal compartments is a major limiting step [13]. More recent quantitative analyses confirmed that only a small fraction of internalized material reaches the cytosol, even when cellular uptake is efficient [50].

Mechanistic reviews across delivery platforms identify shared principles governing endosomal escape. These include ionizable lipid protonation, membrane destabilization, and pore formation [72,73]. However, escape efficiencies remain low and variable between systems. Increasing hepatocyte uptake alone is therefore insufficient without parallel strategies to enhance productive intracellular release [50]. Technologies designed to disrupt endosomal membranes offer potential solutions. Each approach presents trade-offs between activity and tolerability. No broadly applicable clinical solution has yet emerged [72,74].

For approaches targeting nuclear viral templates, additional constraints apply. Nuclear entry of large nucleic acid cargos is inefficient in nondividing hepatocytes. This limitation restricts strategies that rely on direct nuclear import. Evidence suggests that smaller effectors or modalities that act indirectly on nuclear targets may offer more practical paths forward [75,76]. Progress in this area depends on reliable quantification of intracellular routing. Advanced imaging and biochemical assays now provide more accurate assessments of intracellular delivery. These tools increasingly guide rational platform design [13,75].

### 4.6. Translational Constraints and Safety Considerations

Translation of hepatocyte-targeted delivery systems requires careful evaluation of manufacturability, scalability, and long-term safety. Patisiran, the first approved small interfering RNA therapeutic, uses an LNP formulation. This formulation requires intravenous infusion and complex manufacturing. In contrast, GalNAc–small interfering RNA conjugates permit subcutaneous dosing and rely on simpler chemistry. This contrast illustrates how platform choice influences translational feasibility and clinical use [52,77]. These differences also affect formulation reproducibility and scalability in chronic conditions such as hepatitis B.

Beyond route of administration, process complexity, supply-chain stability, and cost of goods influence suitability for widespread use. Reviews consistently show that GalNAc conjugates offer predictable PKs and practical dosing schedules. NP-based systems present greater challenges for repeated outpatient treatment [78,79].

Safety during repeated dosing is a central concern in chronic hepatitis B. LNPs and related carriers can activate innate immune pathways. These effects may not be evident in short-term studies. Extended safety evaluation is therefore essential to identify cumulative toxicity and immunogenicity [80]. Surface chemistry plays a critical role. Some excipients can alter immune recognition during chronic administration. Early integration of repeat-dose toxicology and immunogenicity assessment is necessary for clinical translation.

Regulatory evaluation of advanced delivery technologies increasingly requires clear evidence of target engagement, biodistribution, and sustained KD effects. Quantitative PK and PD analyses are central to demonstrating hepatocyte exposure over time. Advances in imaging and biomarker approaches further support translation of preclinical findings into clinical assessment. These tools enable evaluation of safety and efficacy for first-in-class delivery platforms [79]. The biological and translational constraints discussed in this section are summarized in Table 1.

## 5. Targeted Drug Delivery Strategies Applied to Chronic Hepatitis B Therapy

This section reviews targeted drug delivery strategies according to therapeutic intent. Each subsection focuses on how delivery affects target engagement, intracellular activity, and clinical feasibility across the major classes of therapies under development for chronic hepatitis B.

### 5.1. Improving Delivery of Conventional Antiviral Agents

Nucleos(t)ide analogs remain the clinical foundation for suppressing HBV replication. However, they do not eliminate intranuclear viral templates that sustain antigen production. As a result, their antiviral effect depends on efficient intracellular activation and retention within hepatocytes rather than on liver exposure alone [81,82]. For this therapeutic class, delivery optimization has mainly been achieved through prodrug design. This approach enhances intracellular drug activation within hepatocytes without the need for external carrier systems [83,84].

PK and PD studies show clear differences among prodrugs in their ability to generate active intracellular metabolites in hepatocytes. These differences influence antiviral potency and dosing intervals. For example, tenofovir prodrugs differ in cellular uptake and phosphorylation kinetics. This variation leads to distinct intracellular half-lives of the active metabolite [82,85].

Tenofovir alafenamide represents a clinically validated example of delivery-driven optimization. Its phosphoramidate structure promotes preferential uptake and intracellular activation in hepatocytes. This results in higher intrahepatic concentrations of tenofovir diphosphate. It also leads to lower systemic exposure compared with tenofovir disoproxil fumarate [83,84,86]. Importantly, this targeted intracellular activation improves renal and bone safety. It preserves antiviral efficacy and the underlying mechanism of viral inhibition [87,88].

Additional delivery strategies have been explored to further enhance hepatocyte exposure or prolong intracellular retention. These include modified prodrug chemistry and NP-based formulations designed to increase parenchymal delivery. Experimental studies have evaluated nanoparticle and long-acting formulations to extend hepatic exposure and dosing intervals. However, these approaches remain preclinical. They have not demonstrated clear clinical advantages over optimized oral prodrugs in chronic hepatitis B [89,90]. Despite optimized intracellular activation, nucleos(t)ide analog prodrugs do not eradicate cccDNA. They rarely induce functional cure. As a result, durable off-treatment control remains uncommon [91,92].

### 5.2. Targeted Delivery of Nucleic Acid–Based Therapeutics

Nucleic acid therapeutics, including small interfering RNAs and antisense oligonucleotides, directly reduce viral transcripts and antigen production when delivered effectively to hepatocytes. Their clinical potential depends on three linked delivery requirements: stability in circulation, selective hepatocyte uptake, and productive intracellular release [93,94]. Unlike small-molecule antivirals, these agents cannot cross cellular membranes on their own. As a result, delivery system design is essential for therapeutic activity in vivo [14,95].

Receptor-mediated targeting using GalNAc conjugation has successfully translated to the clinic. This strategy enables efficient hepatocyte delivery after subcutaneous administration. Early clinical and translational studies have reported sustained reductions in HBV RNA and HBsAg following GalNAc-conjugated small interfering RNA therapy [93,94].

These results demonstrate that delivery efficiency strongly determines biological effect. Structurally, GalNAc conjugates rely on multivalent carbohydrate ligands that bind the asialoglycoprotein receptor. This receptor is highly expressed on hepatocytes and undergoes rapid recycling. These features support efficient receptor-mediated endocytosis [55,56].

Despite efficient hepatocyte uptake, key intracellular barriers remain. Endosomal sequestration limits the fraction of internalized oligonucleotides that reach the cytosol. Intracellular processing can also shorten the duration of activity. As a result, repeat dosing is often required. Quantitative imaging studies and mechanistic analyses consistently identify endosomal escape as the primary intracellular bottleneck for nucleic acid therapeutics [13,96]. These studies show that only a small proportion of internalized material escapes endosomes, even when cellular uptake is efficient.

Current development efforts, therefore, combine hepatocyte targeting with strategies to enhance endosomal release or prolong intracellular persistence. Approaches under investigation include optimization of oligonucleotide chemistry, use of ionizable lipid components, and structural modifications that improve membrane interaction in acidic endosomal compartments [72]. The clinical progress of HBV-directed small interfering RNAs highlights the practical impact of these delivery design choices [93]. As a consequence, even highly efficient hepatocyte uptake does not consistently yield sustained antigen reduction. Limited endosomal escape necessitates repeated dosing [61].

### 5.3. Delivery Considerations for Emerging Antiviral Modalities

Capsid assembly modulators and entry inhibitors represent mechanistically distinct antiviral classes. They therefore impose different delivery requirements. Capsid assembly modulators are small molecules with intracellular targets. Their antiviral activity depends on consistent hepatocyte exposure and sufficient cytosolic concentration to disrupt capsid dynamics. Preclinical and early clinical studies demonstrate potent antiviral effects. However, pharmacokinetics and intrahepatic distribution strongly influence response magnitude and durability [97,98].

For capsid assembly modulators, delivery optimization focuses on achieving stable hepatic exposure rather than active cell-specific targeting. These compounds reach their intracellular site of action mainly through passive diffusion and accumulation within hepatocytes [99,100]. Clinical studies indicate that variability in systemic and hepatic exposure can contribute to differences in antiviral response and durability. These findings underscore the importance of PK control for this class [101].

Entry inhibitors, including sodium taurocholate cotransporting polypeptide–targeting agents such as bulevirtide and earlier peptides such as Myrcludex B, act at the hepatocyte surface. Their activity depends less on intracellular delivery and more on sustained presence at the sinusoidal interface. Adequate liver distribution is therefore critical. Structural and pharmacological studies show that receptor engagement and local drug concentration determine antiviral efficacy [102,103]. Because these agents block viral entry at the cell surface, their delivery requirements are defined by receptor occupancy and membrane residence time. They are not determined by endosomal escape or intracellular trafficking [11,12].

Delivery considerations are also central to combination strategies. When agents with different intracellular requirements are combined, hepatic distribution and dosing schedules must be coordinated. This coordination helps ensure that complementary mechanisms act within the same hepatocyte populations [97,98]. Differences in PKs and tissue exposure between entry inhibitors and intracellular antivirals may limit additive or synergistic effects. This highlights the need to align delivery and dosing strategies during regimen design [12,100].

### 5.4. Targeted Delivery of Immune-Modulating Therapies

Immune-modulating therapies aim to restore durable antiviral responses. However, they pose distinct safety challenges. Systemic immune activation can cause collateral liver injury. Localizing immune modulation to the liver or to specific intrahepatic cell populations may enhance antiviral efficacy while limiting systemic toxicity [104]. This issue is particularly important in chronic hepatitis B. Liver injury is largely immune-mediated, and excessive systemic immune activation can worsen hepatic inflammation rather than improve viral control [38].

Delivery platforms that concentrate immunomodulatory agents in the liver or promote uptake by antigen-presenting cells in situ are therefore attractive. At the same time, the liver’s tolerogenic environment and complex immune regulation require precise control. Ongoing preclinical and clinical studies are exploring intraparenchymal delivery, controlled release systems, and cell-directed targeting to balance antiviral benefit with acceptable safety profiles [104,105].

Preclinical studies indicate that restricting immune stimulation to the liver can enhance local antiviral immune responses. These approaches also reduce systemic cytokine exposure. These findings support targeted delivery as a strategy to improve safety rather than to increase immune potency [106].

Delivery strategies must therefore align with therapeutic intent. Modalities acting on intracellular viral templates require hepatocyte specificity and effective intracellular access. Surface-acting agents require sustained exposure at the liver interface. For immune-modulating therapies, spatially restricted delivery functions primarily as a safety-enabling approach. It allows immune activation to be localized and controlled in a disease that requires a careful balance between antiviral efficacy and liver injury risk [38,106]. However, the therapeutic window for immune modulation remains narrow. Even liver-restricted delivery does not fully eliminate the risk of immune-mediated liver injury [107].

**Figure 4:**
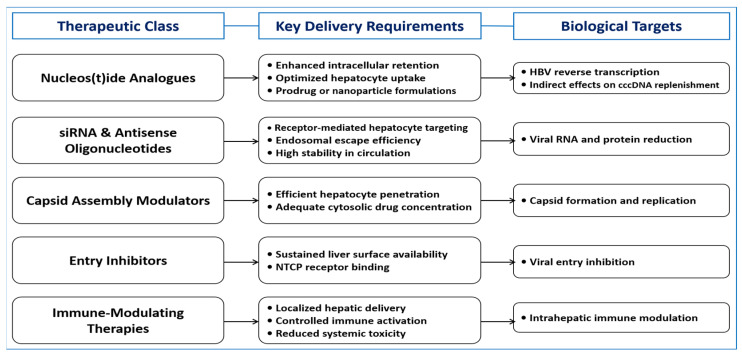
Mapping therapeutic classes to delivery requirements and biological targets in chronic hepatitis B. This schematic illustrates how major classes of antiviral and immune-modulating therapies differ in their delivery requirements according to mechanism of action and site of activity. Nucleos(t)ide analogs, nucleic acid-based therapies, capsid assembly modulators, entry inhibitors, and immune-modulating approaches are aligned with the key delivery constraints that govern hepatocyte exposure, intracellular availability, and safety. The figure emphasizes that effective therapy depends on matching the delivery strategy to the biological target, with distinct requirements for intracellular, membrane-associated, and immune-mediated mechanisms.

Figure 4 illustrates the relationship between therapeutic class, delivery requirements, and biological targets. Table 2 provides an expanded overview of representative hepatocyte-targeted delivery strategies, including clinical development stage, patient populations, key reported outcomes, and major limitations.

## 6. Targeting Viral Reservoirs: Delivery Challenges in cccDNA-Directed Strategies

Achieving a functional cure for chronic hepatitis B requires addressing the central viral reservoir, cccDNA, within the hepatocyte nucleus. cccDNA persists as a chromatinized minichromosome that is tightly associated with host histones and nuclear proteins. This organization regulates viral transcription and stabilizes the episome. At the same time, it limits direct access to the viral template. As a result, strategies designed to silence or eliminate cccDNA face substantial biological and delivery barriers [21,118]. This section summarizes the principal delivery challenges that constrain cccDNA-directed therapies. It also explains why delivery, rather than molecular design, often determines clinical feasibility.

### 6.1. Nuclear Localization and Chromatin-like Organization of cccDNA

cccDNA does not exist as naked viral DNA within the nucleus. Instead, it is organized into a minichromosome that closely resembles host chromatin. Transcriptional activity depends on nucleosome positioning and epigenetic regulation. Experimental studies show that chromatin regulators strongly influence viral transcription. They also restrict access of enzymes and nucleic acid-based effectors to the cccDNA template [21,119].

This nuclear organization has direct implications for delivery. Nuclear import is inefficient in nondividing hepatocytes. Large ribonucleoprotein complexes or genome-editing cargos cross the nuclear pore poorly [120]. Even when nuclear entry occurs, chromatin structure can limit guide binding and nuclease access to target sequences. Experimental use of chromatin-modifying agents can improve accessibility. However, these approaches raise safety concerns and complicate clinical translation [119,121].

### 6.2. Delivery Constraints for Genome-Editing and Epigenetic Approaches

Genome-editing and epigenetic strategies are under development to achieve durable suppression or elimination of HBV cccDNA. These include CRISPR-based nucleases, base editors, prime editors, and epigenetic effectors. Some repress cccDNA transcription without inducing DNA cleavage. Each modality imposes specific delivery requirements. Active components must reach infected hepatocytes. They must access the appropriate intracellular compartment. They must also retain sufficient activity to act on cccDNA or its transcriptional regulation [122,123].

Cargo size and composition represent major constraints. Cas proteins and many editing systems are large and difficult to package into clinically acceptable carriers. Viral vectors such as adeno-associated virus can deliver smaller editors. However, payload capacity is limited, and concerns related to immunogenicity and genomic integration persist. Nonviral systems can accommodate larger cargos. In practice, they often lack sufficient efficiency and durability in vivo [120,124].

To address these limitations, emerging non-viral platforms such as virus-like particles and polymer-based carriers are being explored. These systems aim to improve intracellular delivery and nuclear access while reducing immunogenicity associated with viral vectors. Their efficiency and scalability remain under active investigation [125,126,127].

Cellular entry and endosomal escape impose additional barriers. Many delivery platforms rely on endocytosis. Only a small fraction of internalized material escapes into the cytosol. Quantitative imaging and mechanistic studies consistently identify endosomal escape as a rate-limiting step for functional activity. This limitation directly reduces the amount of active agent that can reach the nucleus [128,129].

After cytosolic release, nuclear access remains inefficient in quiescent hepatocytes. Strategies that rely on cell division to facilitate nuclear entry are not applicable in adult liver tissue. Direct nuclear targeting approaches have shown limited success to date [120].

Epigenetic approaches face related constraints. Although some epigenetic editors are smaller than nucleases, they must persist for sufficient duration or be administered repeatedly. This persistence is required to remodel cccDNA-associated chromatin. Repeated dosing increases the risk of immune activation and cumulative toxicity. These factors limit long-term feasibility [21,130].

### 6.3. Safety, Specificity, and Durability Concerns Tied to Delivery

Delivery strongly influences off-target exposure, immune activation, and durability of response. Many carriers activate innate immune pathways. These responses can reduce efficacy or restrict repeat dosing. LNPs and viral vectors trigger immune responses through distinct mechanisms. In chronic hepatitis B, where prolonged or combination therapy is anticipated, these immune constraints are particularly relevant [128,131].

Specificity is a central concern. Inefficient targeting increases exposure of nonhepatic tissues and uninfected liver cells. Off-target DNA modification remains one of the most serious risks associated with genome editing. Such activity depends on editor design, guide sequence, and chromatin context. Delivery strategies that concentrate active agents within infected hepatocytes and the correct intracellular compartment reduce systemic exposure. They also lower the risk of unintended edits [131,132].

Durability also remains difficult to achieve. Effective cccDNA silencing requires either highly efficient single exposure or repeat dosing that is safe and sustainable. Viral vectors may provide durable expression. However, they raise concerns related to integration and immune responses. Nonviral systems typically provide transient delivery. They therefore require repeat administration, increasing cumulative risk. To date, no delivery platform has combined high efficiency, hepatocyte specificity, reliable nuclear access, and acceptable safety for chronic use [124,128].

### 6.4. Why Delivery, Rather than Molecular Design, Often Limits Clinical Feasibility

Molecular editing tools have advanced rapidly. Improvements in guide design and editor fidelity have reduced intrinsic off-target activity. However, clinical success depends on effective delivery to cccDNA in vivo. Delivery remains limiting for three primary reasons.

First, delivery barriers are sequential. A carrier must survive systemic circulation. It must cross the sinusoidal endothelium. It must avoid sequestration by nonparenchymal cells. It must enter hepatocytes, escape endosomes, and reach the nucleus. Failure at any step prevents effective activity [120,128].

Second, safety scales with dose and tissue distribution. Inefficient delivery requires higher systemic doses to achieve nuclear activity. Increased dosing amplifies immune activation and off-target exposure [128,131].

Third, clinical translation demands scalable and repeatable platforms. Some delivery systems perform well in experimental models but fail to meet manufacturing, regulatory, or cost requirements for human use. GalNAc conjugation illustrates a translatable platform. It combines hepatocyte specificity with predictable PKs and scalable manufacturing. Platforms lacking these attributes frequently fail before clinical validation [118,132].

### 6.5. Practical Directions to Prioritize

Current evidence supports a pragmatic strategy. Hepatotropic delivery systems with demonstrated clinical feasibility should be prioritized. This approach limits systemic exposure and permits infrequent dosing. GalNAc-conjugated RNA interference therapies have produced reductions in viral markers in clinical and translational studies. These systems are well suited to pair with approaches that do not require direct nuclease access to compact nuclear chromatin. Examples include RNA interference to reduce antigen levels and epigenetic silencing of cccDNA with minimized genotoxic risk [93,94,133].

When direct genome editing is pursued, emphasis should be placed on compact, high-precision editors. Delivery systems should be optimized for endosomal escape and nuclear access. Rigorous assessment of off-target activity and immune responses should be integrated early in development [120,128,132].

cccDNA persists as a chromatinized minichromosome within the hepatocyte nucleus. This organization explains both the rationale for direct targeting and the difficulty of achieving it in chronic hepatitis B. Although advances in editor design have reduced biochemical risk, delivery remains the dominant barrier to clinical translation. Access to nuclear cccDNA and immune constraints within hepatocytes limit effective targeting [21,134].

Progress will depend on platforms that combine efficient hepatocyte targeting, productive intracellular trafficking, controlled nuclear access, and acceptable safety during repeat dosing. Until such systems mature, combination strategies that reduce antigen production through cytosolic mechanisms and restore immune control represent the most realistic path toward a functional cure [21,120,128].

## 7. Translational and Clinical Considerations

This section bridges experimental promise and clinical implementation. It highlights the major translational gaps that limit the progression of hepatocyte-targeted delivery strategies from preclinical studies to real-world application in chronic hepatitis B.

### 7.1. Differences Between Preclinical Models and Human Infection

Preclinical models differ from human chronic hepatitis B in several critical aspects. Many cell lines and animal models do not accurately reproduce human hepatocyte biology, cccDNA dynamics, or the immune environment of chronic infection. Primary human hepatocytes and humanized liver models more closely reflect human disease. However, they remain limited in scalability and throughput. These limitations can generate both false-positive and false-negative signals for delivery efficiency and antiviral efficacy. Validation of delivery concepts across complementary platforms is therefore essential before clinical translation. These platforms include primary human cells and relevant animal models [135,136].

Rodent and nonhuman models also differ from humans in liver architecture, receptor expression, and clearance pathways. These differences can lead to overestimation of hepatocyte uptake and underestimation of immune-mediated clearance in humans [54,59]. Early testing in human-relevant systems and conservative extrapolation from animal data reduce the risk of translational failure [137].

### 7.2. Scalability and Manufacturability of Delivery Systems

Platform selection strongly influences translational feasibility. GalNAc-conjugated oligonucleotides are chemically defined and readily scalable for subcutaneous administration. In contrast, LNP systems require more complex formulation, quality control, and cold-chain logistics. These requirements increase manufacturing burden. Adeno-associated viral vectors and other viral platforms present distinct chemistry, manufacturing, and control challenges. They also have inherent capacity limitations. Early assessment of process complexity, cost of goods, and supply-chain risk is therefore critical [62].

Manufacturing complexity also affects dosing logistics, cost, and geographic accessibility. Chemically defined conjugates typically scale more efficiently than multicomponent NP systems. They are often better suited for chronic outpatient treatment [52,138]. Supply chain constraints and stability requirements further limit rapid scale-up and distribution of some platforms. These challenges are particularly relevant in low-resource settings [139].

### 7.3. Regulatory Considerations for Advanced Delivery Technologies

Regulatory agencies require clear nonclinical evidence of biodistribution, target engagement, and safety. Guidance documents for gene and advanced therapies emphasize comprehensive biodistribution assessment and long-term follow-up. For novel delivery platforms, regulators expect robust chemistry, manufacturing, and control data. They also require validated analytical methods and a strategy for monitoring off-target exposure. Early interaction with regulatory authorities can accelerate development. It can also clarify data expectations for first-in-human studies [140,141,142].

Regulatory guidance increasingly calls for integrated PK, PD, and biomarker data. These data are needed to support chronic or repeat dosing. Long-term monitoring plans are also required to assess durability of effect and delayed adverse outcomes [140,143].

### 7.4. Long-Term Safety and Repeat-Dose Requirements

Chronic hepatitis B is likely to require prolonged or repeated treatment. Repeat dosing introduces distinct safety considerations. LNPs and some formulation components can activate innate or adaptive immune responses upon re-exposure. Anti–polyethylene glycol and anti-carrier antibodies have been reported. These antibodies may alter PKs or safety on subsequent dosing. Clinical programs must therefore incorporate repeat-dose toxicology and immune monitoring from early development stages [144,145,146].

Repeated exposure to polyethylene glycol-modified or NP-based carriers can elicit antibodies that change clearance. In some cases, this can trigger complement activation or inflammatory reactions upon re-dosing. Monitoring for these immune responses is essential in chronic treatment settings [144,147,148]. Platforms that enable lower effective doses or longer dosing intervals reduce cumulative exposure. They may therefore offer improved safety profiles for long-term use [149].

### 7.5. Practical Trial and Implementation Issues

Early clinical studies should prioritize measurement of biodistribution, target engagement, and relevant pharmacodynamic markers within the liver. Biomarkers that reflect hepatocyte exposure and antigen reduction are often more informative than plasma PKs alone. Patient selection also influences outcomes. Background nucleos(t)ide analog therapy, fibrosis stage, and baseline antigen levels affect both safety and efficacy readouts. Consideration of affordability and global access is essential. The burden of HBV is highest in low- and middle-income countries [150,151].

Combination regimens are emerging as the most promising therapeutic approach. Clinical trials should therefore plan coordinated dosing strategies and harmonized safety monitoring. They should also incorporate biomarker frameworks that capture both antiviral and immune effects [152].

Global access considerations further support prioritization of platforms that allow scalable manufacturing and outpatient administration. These features maximize impact in high-burden settings [3]. Table 3 summarizes the major translational and clinical challenges that limit implementation of hepatocyte-targeted delivery strategies for chronic hepatitis B, together with their impact on development and key mitigation considerations.

## 8. Future Perspectives: Integrating Biology and Delivery Toward a Functional Cure

This section integrates insights from virology, immunology, pharmaceutics, and clinical translation. It outlines realistic directions for advancing hepatitis B cure strategies while accounting for biological complexity and delivery constraints.

### 8.1. Need for Combination Therapies and Coordinated Delivery Strategies

Monotherapy has limited capacity to eliminate cccDNA or reverse immune exhaustion. Clinical studies and recent reviews consistently report greater antigen reduction and higher rates of functional cure when antiviral silencing is combined with immune modulation. RNA interference and direct-acting antivirals reduce viral antigen levels. Immune-modulating agents promote recovery of virus-specific immune responses. Combining these mechanisms increases the likelihood of durable viral control [93,153,154].

Coordinated delivery is therefore essential. Delivery systems must align with each agent’s biological target, intracellular site of action, and dosing requirements. Hepatotropic GalNAc conjugates are well suited for repeated RNA interference dosing. Other modalities, including immune modulators or genome-editing approaches, may require distinct delivery platforms to reach their intended site of action. Designing combinations with compatible delivery profiles reduces safety risk and improves clinical feasibility [155,156].

### 8.2. Disease-Stage Specific and Patient-Tailored Approaches

Chronic hepatitis B is a heterogeneous disease. Patients differ in viral load, cccDNA burden, liver fibrosis stage, and the extent of viral DNA integration. Clinical guidelines and recent reviews emphasize tailoring therapy according to disease stage and host characteristics. Early stage disease may respond best to antigen reduction combined with immune priming. In contrast, advanced disease or extensive viral integration may require prolonged viral suppression and focused cancer surveillance. Clinical trials should stratify patients and report outcomes by disease stage to support meaningful interpretation [8,157].

Biomarkers are central to personalization. Host and viral markers can help identify patients likely to respond to antigen reduction. They can also identify those requiring stronger immune intervention or those able to tolerate repeat dosing. Integrating biomarker development into clinical trials supports rational, patient-centered treatment strategies [93,158].

### 8.3. Integration of Virology, Immunology, and Pharmaceutics

Progress toward a functional cure requires coordinated input across disciplines. Virology defines the relevant viral targets. Immunology defines functional and durable endpoints. Pharmaceutics ensures that active agents reach the correct cells and intracellular compartments. Recent analyses highlight the need for integrated hepatitis B research programs that link these fields rather than advancing them in isolation. Early sharing of data on biodistribution, intracellular activity, and immune effects accelerates translation from mechanism to clinical outcome [153,159].

In practice, this integration requires coordinated development pipelines. Antiviral and immune-based therapies should be evaluated together in human-relevant models. Delivery scientists should participate in early development decisions. First-in-human studies should collect linked PK, PD, and immune data. These measures reduce late-stage failure and inform rational dose selection and treatment sequencing [160].

### 8.4. Key Knowledge Gaps and Research Priorities

Several priorities warrant focused attention. First, improved human-relevant models are needed. Current systems do not reliably predict human delivery efficiency or immune responses. Expansion of scalable humanized liver models and organoid platforms will support evaluation of delivery alongside viral and immune outcomes [160].

Second, quantitative delivery metrics require standardization. Measurements of hepatocyte exposure, endosomal escape, and nuclear access should become routine. Imaging and tissue pharmacology tools can provide these data and guide platform selection [93,155].

Third, durable and safe repeat dosing remains a major challenge. Delivery systems should enable low-frequency dosing or low cumulative exposure while maintaining activity. This approach reduces immunogenicity and cumulative toxicity. Comparative studies examining dosing intervals and carrier-associated immune effects are needed [155,156].

Fourth, clinical trial designs must evolve to support combination strategies. Adaptive and staged trials can evaluate treatment sequence, timing, and biomarker-guided patient selection. Such designs will clarify which combinations achieve sustained antigen loss and immune recovery [154,161].

Overall, a functional cure for hepatitis B is achievable when biological insight is aligned with delivery and clinical realities. Progress will depend on rational combination therapies, patient-tailored strategies, and delivery platforms that are effective, safe, and scalable. Emphasis on human-relevant validation, interdisciplinary collaboration, and pragmatic trial design offers the most reliable path toward durable clinical benefit [153,158].

## 9. Conclusions

Chronic hepatitis B remains difficult to cure because viral persistence is maintained within hepatocytes through cccDNA and integrated viral DNA, which are not eliminated by current antiviral therapies. Although existing treatments effectively suppress viral replication, sustained antigen loss and durable functional cure remain uncommon. This review shows that drug delivery to hepatocytes is a central determinant of therapeutic success, not a secondary technical issue. Liver exposure alone is not enough. Effective therapies must reach infected hepatocytes, move productively within cells, and, for some approaches, access nuclear viral templates. The structure and biology of the liver create sequential barriers that greatly reduce the amount of drug reaching its intended intracellular target. Recent clinical progress with hepatocyte-targeted delivery, especially ligand-based systems such as GalNAc conjugation, demonstrates that precise, scalable hepatocyte targeting is achievable in humans and can produce meaningful reductions in viral transcripts and antigens. In contrast, more complex delivery platforms still face major challenges related to intracellular access, immunogenicity, scalability, and repeat-dose safety. These limitations are most evident for strategies aimed at nuclear viral reservoirs, where delivery constraints, rather than molecular design, currently limit clinical feasibility. Overall, the evidence supports a pragmatic path toward functional cure based on combination strategies that reduce antigen burden and restore antiviral immune control, rather than reliance on single-modality approaches. Progress will depend on aligning viral biology with delivery feasibility, clinical safety, and patient heterogeneity, with delivery science positioned at the core of therapeutic development.

## Figures and Tables

**Table 1:** Key biological and translational principles guiding hepatocyte-targeted drug delivery in chronic hepatitis B.

Barrier or Design Principle	Biological Basis in the Liver	Implications for Hepatocyte Delivery	Representative Strategies or Examples	References
Hepatocyte specificity	HBV persistence and transcriptional templates are confined to hepatocytes	Liver exposure alone does not ensure antiviral efficacy	Hepatocyte-directed targeting strategies	[51,52]
Receptor-mediated uptake	ASGPR is highly expressed on hepatocytes and undergoes receptor recycling	Uptake efficiency depends on receptor availability and dosing	Multivalent GalNAc conjugation	[55,56,59]
Ligand design constraints	Ligand affinity and valency influence receptor engagement and intracellular routing	Excessive affinity or dose may cause transient receptor saturation	Geometry-controlled GalNAc displays; peptide-based ligands	[54,59]
Sinusoidal fenestration	Endothelial fenestrae restrict size-dependent access to the parenchyma	Large particles show reduced hepatocyte exposure	Size-controlled NPs	[64,65]
Protein corona formation	Serum protein adsorption promotes recognition by Kupffer cells	Off-target uptake reduces parenchymal bioavailability	Surface chemistry modulation; biomimetic coatings	[67,68]
Surface charge and ionizability	Surface charge affects circulation, uptake, and intracellular processing	Strongly cationic surfaces increase toxicity risk	Ionizable lipid formulations	[46,63]
Non-parenchymal sequestration	Kupffer cells and liver sinusoidal endothelial cells efficiently clear particulates	Significant dose loss occurs before hepatocyte engagement	Stealth coatings; ligand-mediated redirection	[67,71]
Endosomal escape	Internalized cargo is frequently retained within endosomes	Cellular uptake does not guarantee functional delivery	Ionizable lipids; endosomolytic strategies	[13,50]
Nuclear access constraints	Large nucleic acids poorly access the nucleus in nondividing hepatocytes	Direct nuclear targeting is inefficient	Indirect or cytosolic-acting modalities	[75,76]
Repeat-dose safety	Chronic administration may reveal immune or toxic effects	Short-term studies may underestimate risk	PK/PD-guided dosing; chronic safety assessment	[57,80]
Translational feasibility	Manufacturing complexity and scalability vary across platforms	Platform choice affects long-term clinical use	GalNAc conjugates versus LNPs	[52,78]

**Table 2:** Representative hepatocyte-targeted drug delivery strategies for chronic hepatitis B, including delivery approaches, clinical development status, patient populations, key reported outcomes, and major limitations.

Therapeutic Class	Delivery Strategy	Representative Agent(s)	Clinical Phase/Status	Patient Population	Key Reported Outcomes	Main Limitations	References
Nucleos(t)ide analogs	Hepatocyte-preferential prodrug activation	Tenofovir alafenamide (TAF)	Approved	Treatment-naïve and treatment-experienced CHB patients	Durable suppression of serum HBV DNA; improved renal and bone safety compared with TDF	Does not eliminate cccDNA; HBsAg loss remains uncommon	[86,87,88]
siRNA therapeutics	GalNAc–ASGPR targeting	JNJ-3989 (ARO-HBV)	Phase II	Virally suppressed and treatment-naïve CHB patients	Reductions in HBV RNA and HBsAg observed during active treatment	Rebound of viral markers after treatment discontinuation; repeat dosing required	[9,108]
Antisense oligonucleotides	GalNAc conjugation	GSK3228836 (IONIS-HBV-LRx)	Phase II	HBeAg-positive and HBeAg-negative CHB patients	Dose-dependent HBsAg declines in a subset of patients	Heterogeneous response; injection-site reactions and immune-mediated ALT flares reported	[9]
Capsid assembly modulators	Oral small-molecule delivery	JNJ-56136379, ABI-H0731	Phase II (modified or discontinued)	CHB patients on NA therapy	Suppression of HBV DNA and RNA when administered in combination with nucleos(t)ide analogs	Limited effect on hepatitis B surface antigen; pharmacokinetic variability; resistance concerns; several programs modified or discontinued	[10,100]
Entry inhibitors	NTCP receptor blockade	Bulevirtide (Myrcludex B)	Approved for HDV; Phase II for HBV	HBV/HDV co-infected and HBV mono-infected patients	Effective inhibition of viral entry; robust suppression of HDV replication	Minimal effect on hepatitis B surface antigen; long-term administration required	[109,110]
Immune-modulating therapies	Systemic or liver-biased delivery	TLR agonists; therapeutic vaccines	Phase I–II	Virally suppressed CHB patients	Immune activation observed in early-phase studies; limited and inconsistent antiviral effects	Safety concerns related to immune-mediated liver injury; transient responses	[111,112,113]
Genome-editing approaches	Viral and non-viral delivery systems	CRISPR/Cas-based editors	Preclinical	Not applicable	Efficient cleavage and silencing of HBV DNA and cccDNA demonstrated in cell culture and animal models	Delivery efficiency, long-term safety, off-target effects, and durability of response not established in humans	[114,115,116,117]

Footnote: Reported outcomes primarily reflect on-treatment effects unless otherwise specified. Listed limitations highlight current gaps in durability, safety, and delivery efficiency. Approaches that remain at the preclinical stage are explicitly indicated where clinical data are not available.

**Table 3:** Translational and clinical challenges in HBV-targeted drug delivery.

Translational Challenge	Impact on Clinical Translation	Affected Delivery Platforms	Key Considerations and Mitigation Strategies	References
Differences between preclinical models and human infection	Overestimation of efficacy and hepatocyte exposure in humans	All delivery platforms	Use primary human hepatocytes and humanized liver models; interpret animal data conservatively	[54,59,135,136,137]
Limited hepatocyte targeting efficiency	Reduced on-target activity and increased systemic exposure	NPs, viral vectors, conjugates	Prioritize hepatocyte-specific ligands; assess biodistribution early	[62,122,123]
Inefficient intracellular and nuclear access	Low functional activity despite cellular uptake	Genome-editing cargos, epigenetic editors	Optimize endosomal escape; favor modalities with lower nuclear access requirements	[120,128,129]
Scalability and manufacturability constraints	Delayed development and limited global deployment	LNPs, viral vectors	Evaluate chemistry, manufacturing, and controls feasibility early; prioritize scalable platforms	[62,138,139]
Regulatory expectations for advanced delivery systems	Extended timelines and increased data requirements	Gene therapies, novel delivery systems	Generate biodistribution and long-term safety data; engage regulators early	[140,142,143]
Repeat-dose safety and immunogenicity	Loss of efficacy or adverse reactions with chronic dosing	PEGylated systems, NPs, viral vectors	Monitor immune responses; design for lower doses or longer intervals	[20,144,145,147,148,149]
Trial design and biomarker limitations	Inadequate assessment of hepatic target engagement	All delivery platforms	Use liver-specific PD markers	[143,150,151]
Global access and affordability	Limited impact in high-burden regions	Complex or high-cost platforms	Favor outpatient-compatible and scalable systems	[3,149,150]

## Data Availability

No new data were created or analyzed in this study. Data sharing is not applicable.
